# The ProCVT smart-sheet as a new screening tool for cardiovascular autonomic neuropathy: a feasibility study

**DOI:** 10.3389/fendo.2025.1653881

**Published:** 2025-10-21

**Authors:** Maria Bitsch Poulsen, Peter Julu, Johan Røikjer, Asbjørn Mohr Drewes, Christina Brock

**Affiliations:** ^1^ Mech-Sense, Department of Gastroenterology, Aalborg University Hospital, Aalborg, Denmark; ^2^ Department of Clinical Medicine, Aalborg University, Aalborg, Denmark; ^3^ William Harvey Heart Centre, Queen Mary University of London, London, United Kingdom; ^4^ Steno Diabetes Center North Denmark, Aalborg University Hospital, Gistrup, Denmark; ^5^ Department of Endocrinology, Aalborg University Hospital, Aalborg, Denmark

**Keywords:** type 2 diabetes, cardiovascular autonomic neuropathy, parasympathetic nervous system, ECG, cardiac vagal tone

## Abstract

**Introduction:**

Cardiovascular autonomic neuropathy (CAN) is a severe complication of diabetes that impairs the regulation of the cardiovascular system. This can cause hemodynamic instability, arrhythmias, and silent ischemia. Despite its clinical significance, routine testing is not widely implemented. Therefore, this study investigated the performance of a novel screening tool, the *ProCVT* smart-sheet, based on electrocardiography (ECG)-derived cardiac vagal tone (CVT), compared to standardized methods in type 2 diabetes (T2D).

**Methods:**

Forty individuals with T2D (aged 45–75) with varying degrees of CAN and 20 age-matched controls were included in this cross-sectional study. Autonomic profiling included cardiovascular autonomic reflex tests, short-term CVT, 72-hour blood pressure monitoring, and three nights of home monitoring with the *ProCVT* smart-sheet. Receiver operating characteristics assessed the performance of long-term and short-term CVT to detect any, early-stage, and manifest CAN.

**Results:**

A total of 164 recordings were obtained, with an average of 93% of each recording classified as very high signal quality before artifact removal. Short- and long-term mean CVT were the best-performing parameters, identifying any and manifest CAN with AUCs of 0.64–0.79. Suggested cut-offs were 2.7 linear vagal scale (LVS) units for short-term and 5.0 LVS for long-term recordings.

**Conclusion:**

The *ProCVT* smart-sheet offers a feasible, non-invasive alternative to traditional ECGs that rely on surface electrodes. CVT shows promise as a biomarker for identifying manifest CAN in T2D. However, long-term recordings of CVT were not superior to short-term recordings. Further research is warranted to assess its value in the detection of early-stage CAN.

## 1 Introduction

Autonomic neuropathy is an incurable complication of diabetes with multiorgan presentation, including the cardiovascular system, called cardiovascular autonomic neuropathy (CAN). Prevalence estimates of CAN vary widely ranging from 12-73% and it contributes to the excess mortality from cardiovascular diseases (1, 2). The cardiovascular system is regulated bidirectionally by the brainstem, controlled by the sympathetic and parasympathetic nervous systems. When this regulation is impaired, the ability to constantly adapt to the physiological and environmental changes is diminished, potentially leading to hemodynamic instability, arrhythmias, and silent ischemia (3–5). The prevalence of diabetes is increasing at an alarming rate, particularly type 2 diabetes (T2D) (6), and with it the number of complications.

The pathophysiology underlying CAN is that the parasympathetic branch of the autonomic nervous system is initially affected, leading to parasympathetic withdrawal and sympathetic compensation, which ultimately progresses to sympathetic withdrawal (7). The late stage of CAN is considered irreversible due to the nerve damage being too severe at this point (8, 9). This emphasizes that the early stages with parasympathetic withdrawal should be of interest to increase early detection, where lifestyle and cardiovascular risk modifications are still effective (10).

The American Diabetes Association recommend annual assessment of autonomic neuropathy, whereas Danish guidelines only advise evaluation at clinical suspicion (11, 12). Yet, up to 44% of individuals with type 2 diabetes exhibited symptoms of autonomic neuropathy in a Danish study (13), suggesting that many cases may go undetected without routine screening. Nonetheless, clinical implementation of the recommendations remains limited, potentially contributing to underdiagnosis and delayed management.

The current gold standard is a test series assessing the integrity of the brainstem-heart connection, the cardiovascular autonomic reflex tests (CARTs). The primary parasympathetic test is a one-minute cycle of deep breathing, which demonstrates RR-interval variation from respiratory sinus arrhythmia, whereas the RR-interval response to postural change and the Valsalva maneuver is a mixed response of both sympathetic and parasympathetic innervation (14). Measures of heart rate variability, baroreflex sensitivity and 24-hour blood pressure monitoring are also common in the assessment of CAN. However, to advance the detection of early-stage CAN, it may be valuable to explore biomarkers that more specifically reflect parasympathetic innervation e.g., vagal rebound during early heart recovery after exercise or cardiac vagal tone (CVT) (15). CVT is a measure of parasympathetic activity derived from a continuous stream of electrocardiograms (ECGs) as pulse-synchronized phase shifts in consecutive cardiac cycles. The normal resting levels of CVT range from 5–15 on the linear vagal scale (LVS) and CVT has previously been shown to be diminished in type 1 diabetes and related to neuropathy status (16–18).

Unless very complicated, T2D is managed in the primary sector. New screening and diagnostic tools should therefore be easy to use to be successful in a primary care facility with limited specialist and technical expertise available. To this purpose, ProBiometrics has developed the *ProCVT* smart-sheet prototype for screening of dysautonomias such as CAN. The sheet is an ECG-recorder that enables wireless, surface electrode-free, long-term recordings of CVT during sleep, where the parasympathetic tone is at its highest. Thus, this study aimed to investigate whether the *ProCVT* smart-sheet is a reliable screening tool to assess CAN in T2D.

## 2 Methods

### 2.1 Participants

At Aalborg University Hospital, this cross-sectional study investigated 40 participants with T2D aged 45–75 years with a minimum disease duration of 5 years on stable antihyperglycemic and hypertensive medication. To reduce selection bias, participants were consecutively recruited from the outpatient clinic and primary sector to ensure a representative sample across a range of disease severity. Exclusion criteria included body mass index (BMI) >35 kg/m^2^, hemoglobin A1c (HbA1c) ≤48 mmol/mol within the last 12 months, an estimated glomerular filtration rate (eGFR) ≤45 ml/min/1.73m^2^, severe cardiovascular disease (NYHA IV or BP >150/100), or previous bariatric surgery. In addition, 20 age-matched healthy participants were included for comparison. Basic information such as medical history, co-morbidities, prescriptions, and routine biochemistry was collected. To determine the autonomic profile, the participants underwent different objective and subjective autonomic tests in the laboratory and were monitored at home for 72 hours. No test equipment required calibration before use. This study was conducted in accordance with the Declaration of Helsinki and good clinical practice (GCP) guidelines with regular monitoring by the local independent GCP unit. This study was approved by the Medical Research Ethics Committee (No. 2209561) and the Danish Medicines Agency (No. 2022072376). It was conducted between February 2023 and November 2024.

### 2.2 Procedures

#### 2.2.1 Laboratory assessments

Participants were instructed to attend after a fast of 8 hours and to abstain from caffeine and nicotine. A blood and urine sample were obtained for biochemical profiling prior to the autonomic assessments, which was performed once, unless otherwise stated, with a few minutes in between tests. CARTs were performed with the handheld Vagus™ (Medicus Engineering, Aarhus, Denmark), which measures the RR-interval response to postural change (30:15 ratio), deep breathing (expiration: inspiration ratio), and the Valsalva maneuver (RR_IV_: RR_II_ ratio). Age-stratified cut-off values provided by the manufacturer were used to categorize into abnormal/normal test results (19). Early CAN was defined as one abnormal result, and manifest CAN as two or more.

Orthostatic intolerance, which was assessed over ten minutes, is defined as a systolic drop of ≥20 mmHg or a diastolic drop of ≥ 10 mmHg. It was classified as initial hypotension if it occurred within the first minute, classic if it occurred between the first and third minute and delayed if it occurred from the fourth to the tenth minute. Sudomotor function was measured in both hands and feet using the Sudoscan (Impeto Medical, California, San Diego, USA) and averaged between the left and right sides.

To estimate the pupil response to light, pupil diameter (in millimeters) was measured with the handheld PLR^®^-3000 (NeuroOptics, California, USA) three times in a dark environment with minimal light exposure and three times during pen light stimulation. The response to light was determined as the difference between the maximum dilated pupil and the maximum constricted pupil.

The NeuroScope (Neurosentronic Ltd, London, UK) was used to measure real-time ECG-derived CVT with a sampling rate of 5000 Hz, and was combined with the Nexfin^®^ (BMEYE, Amsterdam, The Netherlands) and using VaguSoft software (Neurosentronics Ltd, London, UK) to enable concurrent assessment of cardiac sensitivity to baroreflex. A stable segment with limited blood pressure variation was selected from each participant, and artifacts were removed. CVT is measured in units of a clinically validated atropine-derived linear vagal scale (LVS) (20). The LVS has an absolute zero value equivalent to full atropinization in (21).

The questionnaire Composite Autonomic Symptom Score-31 (COMPASS-31) covers symptoms of orthostatic intolerance, vasomotor, secretomotor, gastrointestinal, bladder, and pupillomotor function. The symptom score was determined using Sletten’s method (22).

#### 2.2.2 72-hour home monitoring

Ambulatory blood pressure was measured with the TM-2430 (A&D Company Limited, Tokyo, Japan), which measured every 15 minutes during the day and every 30 minutes at night. Reverse dippers were identified with ≥10% increase in either systolic or diastolic blood pressure compared to the daytime level. In addition, the ePatch (BioTelemetry Technology, Hørsholm, Denmark) was attached to the chest to record single-lead ECG, and blinded continuous transcutaneous glucose monitoring (Dexcom G6, Dexcom, San Diego, CA) were collected. During sleep in this 72-hour period, the participants were instructed to sleep on the *ProCVT* smart-sheet (ProBiometrics, Kent, UK), which contains six non-contact capacitive ECG-electrodes, and one temperature sensor. The ECG electrodes are rectangular and approximately 15 cm wide making it possible to move during sleep. ECG was sampled at 1000 Hz with 24-bit resolution. This setup was intended to capture up to three sleep recordings per participant, helping to account for night-to-night variation and minimize the impact of single-night anomalies on cardiac vagal tone measurements.

### 2.3 Data assessment

Characteristics of the raw data recorded with the *ProCVT* smart-sheet are presented as the number of data points, the amount of missing data, the signal quality rated from 0-15 (the higher, the better), and the number of adverse device effects. Missing data was calculated based on the average heart rate. If the heart rate is ≥60 beats per minute (bpm), it was assumed that a recording would have at least one R-peak per second. If the heart rate is <60 bpm, the expected number of observations is calculated using the mean heart rate, as the assumption of one observation per second no longer applies. This approach avoided unnecessary complexity for HR ≥60 bpm, where the assumption of one R-peak per second is sufficiently accurate, while ensuring an appropriate adjustment for lower HR values, where missed beats could otherwise lead to data misinterpretation.

### 2.4 Data processing

Prior to analysis, the data recorded with the *ProCVT* smart-sheet were processed according to laboratory standards (16, 17). CVT is calculated on beat-to-beat basis, with each value reflecting a continuous rolling average of 10 preceding data points. To avoid edge artifacts caused by the rolling average the first and last 10 datapoints were excluded. Artifacts was defined as sudden beat-to-beat increases in heart rate >15 bpm. Because artifacts are rarely confined to a single beat, thus each artifact was removed together with the seven following data points and the seven preceding points to account for possible preceding distortions and to limit their effect on the rolling average. The inherent software rates the signal quality of each data point on a scale from 0-15, 15 being the highest, and those with a score <10 was removed. Finally, the proportion of valid data points was calculated, and recordings retaining less than 70% of the original data points were excluded. A good representative recording was manually selected from each participant for final analysis. This selection was based on our knowledge that CVT varies with sleep stages (23) with preference given to recordings showing prominent high broad CVT peaks indicating periods of wakefulness and minimal periods with small frequent peaks reflecting arousal. This approach ensured that recordings with low-quality sleep, associated with lowered cardiac vagal tone, were not used in the determination of cut-off values.

To assess parasympathetic capacity during sleep, we calculated the average absolute difference between the whole night mean CVT and the 20 most extreme values; 10 with elevated CVT and 10 with reduced CVT. This measure was defined as ‘CVT capacity’.

### 2.5 Statistical analysis

Descriptive statistics are expressed according to the distribution of data evaluated using QQ-plots and histograms. Proportional differences between groups were determined with a chi-square test. Non-normally distributed continuous data were compared using the Wilcoxon Rank Sum test or the Kruskal-Wallis test with Benjamini-Hochberg correction. Night-to-night variation in mean CVT was determined using the intraclass correlation coefficient from a linear mixed model with recording number as a fixed effect and individual as the random intercept. The intraclass correlation coefficient was used to quantify the proportion of variance in mean CVT attributable to differences between individuals compared to within-individuals. Receiver Operating Characteristic (ROC) analysis was conducted to assess the diagnostic performance of CVT. The area under the curve (AUC) was used as the primary summary metric, with the optimal cut-off point determined via the Youden Index. AUC values and their corresponding 95% confidence intervals (CIs) were estimated using the DeLong method. Missing values were handled using pairwise deletion. No formal sample size estimation was performed, as the primary aim was to assess feasibility and generate preliminary data on the diagnostic performance. Statistical analysis was performed in Stata Statistical Software: Release 18. College Station, TX: StataCorp LLC) or R (R Core Team (2023) or R (R Core Team (2023). R: A Language and Environment for Statistical Computing. R Foundation for Statistical Computing, Vienna, Austria). The significance level was set at 0.05. Figures were produced in R.

## 3 Results

**Table 1** lists the clinical characteristics of the study population. In general, the individuals with T2D had higher BMI, and both antihypertensive and cholesterol-lowering medications were more frequently used. Regarding the biochemical profile, individuals with T2D had higher HbA1C and fasting glucose levels than the controls. However, the individuals with T2D had lower, cholesterol, high-density lipoprotein cholesterol, and low-density lipoprotein cholesterol levels, indicating routine treatment of risk factors according to national guidelines. However, they had higher triglyceride levels, likely related to metabolic syndrome. No major differences were observed in kidney function, but the albumin/creatinine ratio was slightly elevated in the individuals with T2D. In terms of the autonomic profile, manifest CAN was more common in individuals with T2D, and they presented with higher COMPASS-31 scores, lower CVT, lower cardiac sensitivity to the baroreflex (CSB), and had smaller pupil diameter when dilated, all signs of a dysregulated autonomic nervous system.

**Table 1:** Clinical characteristics.

	Control	Type 2 diabetes	P-value
N	20	40	
Age, median (IQR)	60 (56; 70)	64 (59; 69)	0.256
BMI, median (IQR)	25.3 (23.0; 29.3)	29.9 (26.5; 31.9)	**0.003**
Sex male, n (%)	11 (55%)	26 (65%)	0.453
Disease duration (y), median (IQR)		14 (9;20)	
Complications			
Peripheral neuropathy, n (%)		17 (42.5%)	
Retinopathy, n (%)		1 (2.5%)	
Previous foot ulcer, n (%)		1 (2.5%)	
Medication, n (%)			
Antihypertensives,	9 (45%)	31 (77.5%)	**0.012**
Antithrombotic,	2 (10%)	11 (27.5%)	0.121
Cholesterol-lowering,	8 (10%)	29 (72.5%)	**0.015**
Antihyperglycemics,			
Metformin,		33 (82.5%)	
SGLT2 inhibitors,		19 (47.5%)	
Insulin,		16 (40%)	
GLP-1 analogue,		14 (35%)	
DPP-4 inhibitors,		4 (10%)	
Sulfonylureas,		1 (2.5%)	
Biochemical profile			
HbA1c (mmol/mol)	35 (33; 37)	54 (49.2; 55.8)	**0.000**
Fasting glucose (mmol/l)	5.4 (5.2; 5.8)	8.4 (7.4; 9.3)	**0.000**
Triglycerides (mmol/l)	0.9 (0.7; 1.3)	1.5 (0.9; 2.7)	**0.008**
Cholesterol (mmol/l)	4.7 (3.9; 5.2)	3.7 (3.3; 4.6)	**0.012**
HDL cholesterol(mmol/l)	1.5 (1.3; 1.7)	1.2 (0.9; 1.4)	**0.002**
LDL cholesterol (mmol/l)	2.5 (2.3; 3.3)	1.9 (1.6; 2.4)	**0.001**
eGFR (ml/min/1.73m^2^)	88 (83; 96)	96 (87; 100)	0.121
Albumin/creatinine ratio	10.1 (4.6; 18.0)	14.8 (7.9; 37.4)	0.059
Autonomic profile			
Short-term CVT ^a^ (LVS), median (IQR)	4.2 (3.5; 8.6)	2.6 (1.7; 3.6)	**0.000**
Short-term CSB^b,^ median (IQR)	2.8 (1.9; 5.4)	1.6 (0.9; 2.5)	**0.002**
COMPASS-31, median (IQR)	3.7 (1.1; 6.1)	14.1 (6.3; 28.9)	**0.000**
Reverse dippers^c^, n (%)	0 (0%)	1 (2.8%)	1.000
CAN status^d^, n (%)			
Early CAN	3 (15%)	10 (25%)	0.375
Manifest CAN	1 (5%)	11 (27.5%)	**0.040**
Orthostatic intolerance, n (%)			
Initial	2 (10%)	10 (25%)	0.171
Classic	2 (10%)	4 (20%)	1.000
Delayed	2 (10%)	7 (17.5%)	0.443
Sudomotor function, median (IQR)			
Hands (µS)	74 (48; 79)	62 (51; 73)	0.515
Feet (µS)	82 (77; 85)	82 (76; 85)	0.988
Pupil response^e^			
Pupil diameter dilated (mm)	5.4 (4.6; 5.8)	4.5 (4.0; 5.3)	**0.016**
Pupil diameter constricted (mm)	2.7 (2.4; 3.2)	2.7 (2.3; 3.2)	0.865
Δpupil diameter	2.4 (2.1; 2.9)	1.9 (1.4; 2.3)	**0.001**

IQR, interquartile range; BMI, body mass index, SGLT2 inhibitors, sodium-glucose co-transporter-2 inhibitors. GLP-1, glucagon-like peptide analogues. DPP-4 inhibitors, dipeptidyl peptidase-4 inhibitors; HbA1C, hemoglobin A1c; eGFR, estimated glomerular filtration rate; HDL, high-density lipoprotein; LDL, low-density lipoprotein; CAN, cardiovascular autonomic neuropathy, COMPASS-31, Composite Autonomic Symptom Score-31; CVT, cardiac vagal tone. CSB, cardiac sensitivity to the baroreflex. Data available from N individuals: a=55, b=54, c=57, d=58, e=59. P < 0.05 highlighed with bold text.

### 3.1 Data evaluation of the *ProCVT* smart-sheet recordings

Descriptives of the data recorded with the *ProCVT* smart-sheets are outlined in **Table 2**. In total, 164 recordings were obtained out of 180 possible (91.1%). The recordings were, on average, 7 hours long with about 19,716 data points. However, on average, 20.8% of the data was missing, meaning that approximately 5.5 hours of data were available per night. On average, 93.7% of each recording had a signal quality of 13 or higher on the 1–15 scale, i.e., very high-quality data. This study used five different smart-sheets, of which one failed after being handed out to a participant, resulting in no data being available from that individual. After processing, 36 recordings were excluded, meaning that 128 out of the original 180 (71.1%) recordings were available for analysis. These had, on average, 20,816 data points, and the majority contained >90% of the original data after processing.

**Table 2:** ProCVT Smart-Sheet performance data.

Raw data	
Recordings, n	164
Recording length, mean ± SD	7 h 14 min ± 2 h 8 min
Number of data points per recording, mean ± SD	19,716 ± 9,632
Missing data (%), mean ± SD	20.8 ± 32.4 %
High signal quality, ≥13 (%)*, mean ± SD	93.7 ± 9.9 %
Adverse device effects, n	3

Processed data	
Recordings, n	128
Number of data points per recording, mean ± SD	20,816 ± 8,103
Data preservation level, n	
70-80%	15
81-90%	27
>90%	86

*Signal quality is assessed from 1-15, 15 being the highest possible data quality.

### 3.2 Night-to-night variation in long-term CVT

To assess the night-to-night variation in mean CVT, individuals with only one recording were excluded, resulting in a total of 124 recordings from 46 individuals. The linear mixed model yielded an intraclass correlation coefficient of 0.91, indicating that 91% of the total variance is attributable to between-individual variance. Accordingly, the remaining 9% is due to within-individual variance, i.e., night-to-night variation, which proves high reliability.

### 3.3 Association between long-term CVT and CAN

**Figure 1:**
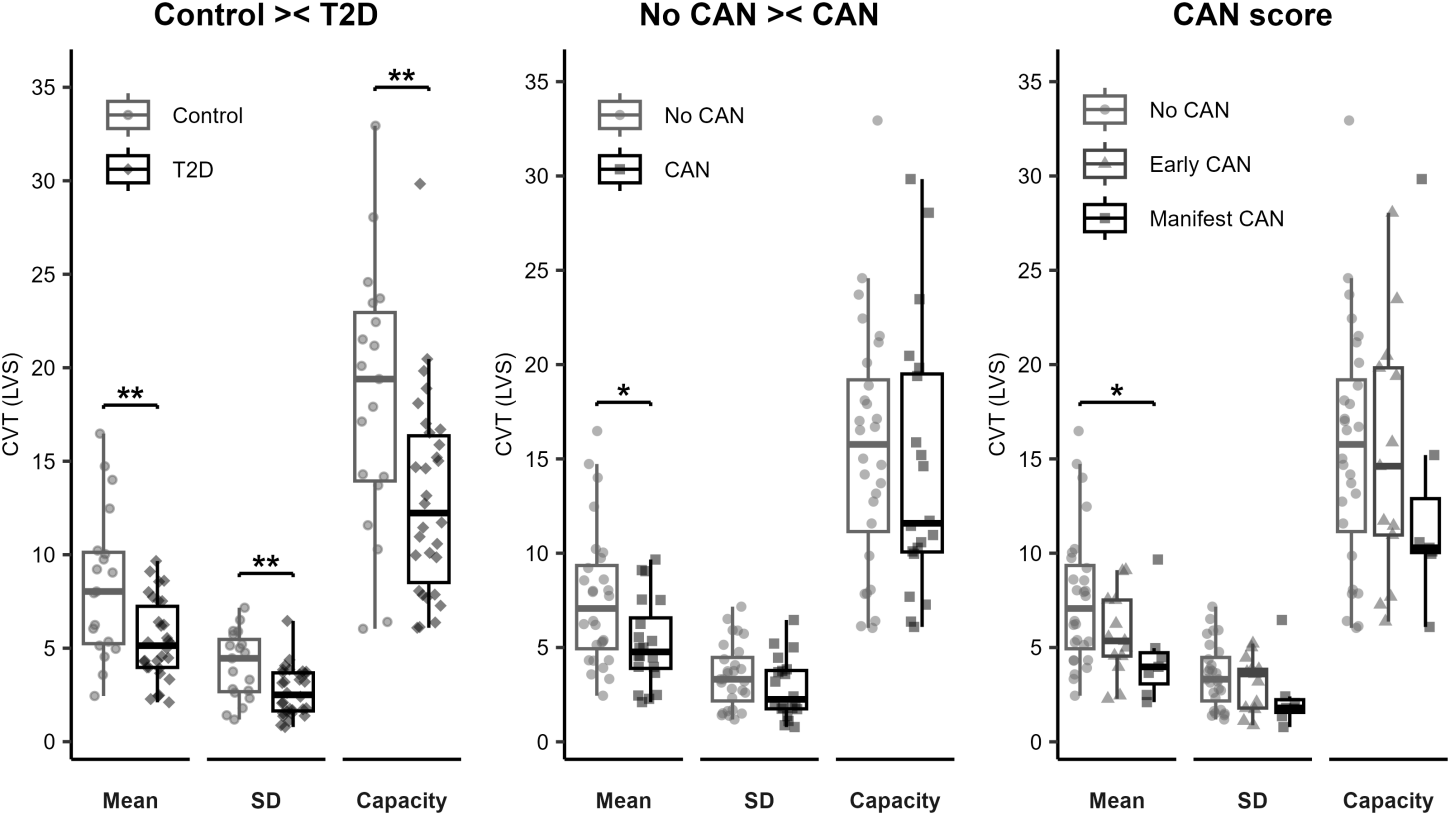
CVT comparisons: control versus individuals with T2D; no cardiovascular autonomic neuropathy versus cardiovascular autonomic neuropathy, and the different stages of cardiovascular autonomic neuropathy. T2D, type 2 diabetes; CAN, cardiovascular autonomic neuropathy; SD, standard deviation. ** indicates P<0.01. * Indicates P<0.05. Data available from 48 participants.

From the 128 recordings available, the best-quality recording from each participant was selected for analysis, resulting in 48 recordings with corresponding CAN status. When comparing the individuals with T2D to the controls, they presented with lower mean CVT (5.2 (4; 7.2) vs. 8 (5.2; 10.1), P = 0.005) with less variation i.e., lower standard deviation (4.5 (2.7; 5.5) vs 2.5. (1;6; 3.7), P = 0.007) and with lower CVT capacity (12.2 (8.0; 16.6) vs. 19.4 (13.7; 23.5), P = 0.007). This is illustrated in **Figure 1**. In the comparison between individuals with any degree of CAN to those without CAN, only the mean CVT was lower (4.8 (3.7; 7.2) vs 7.1 (4.5; 9.6), P = 0.023), and when comparing the three different stages of CAN; those with manifest CAN had lower mean CVT compared to those without CAN (4.0 (2.5; 5.0) vs 7.1 (4.5; 9.6), P = 0.02).

### 3.4 CVT as a biomarker for CAN

**Figure 2:**
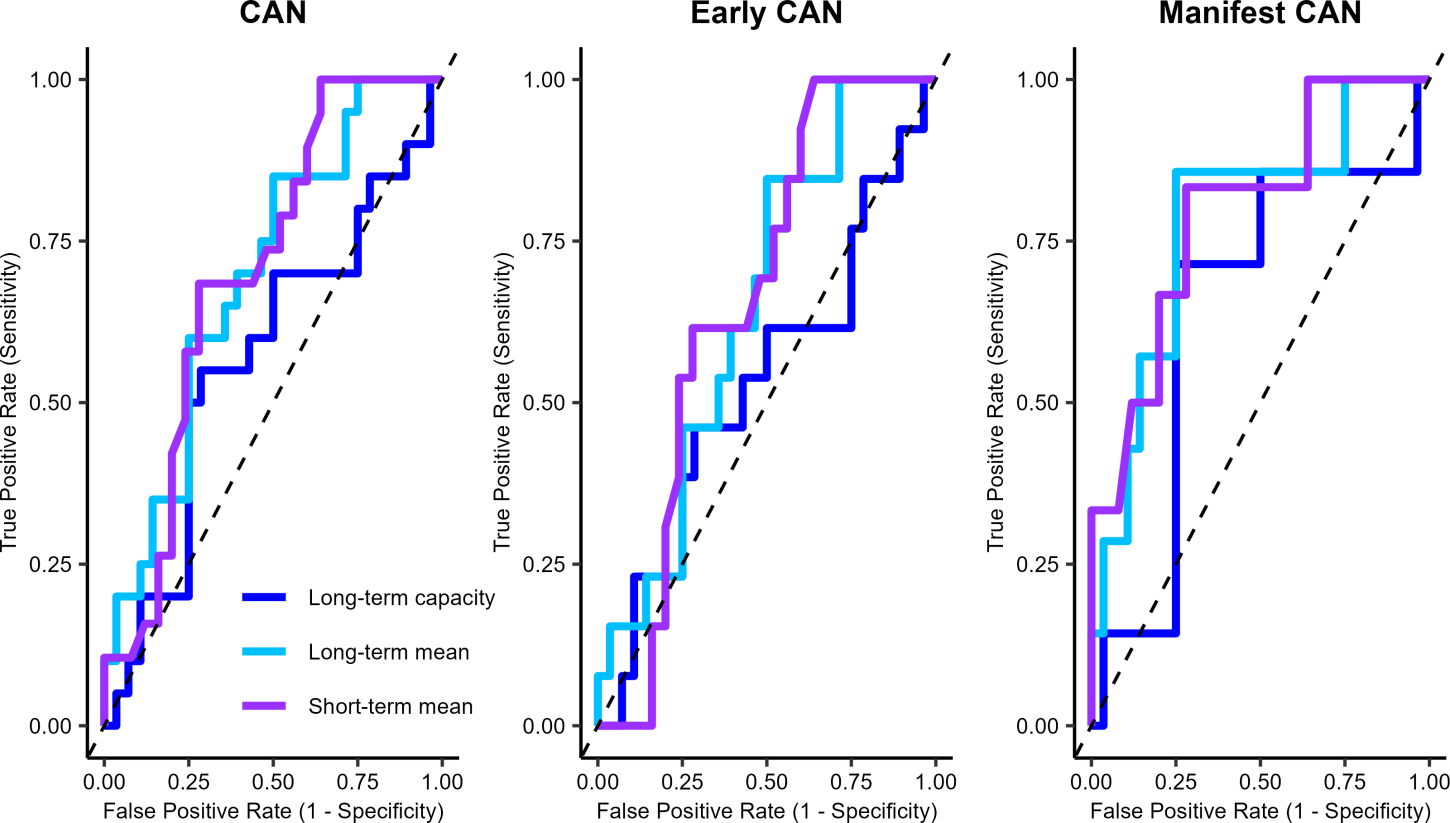
Receiver operating characteristics curve comparing different predictors for cardiovascular autonomic neuropathy based on cardiac vagal tone. CAN: cardiovascular autonomic neuropathy. Number of participants: short-term *N* = 45; long-term *N* = 48.

The above-mentioned CVT measures, along with the short-term mean CVT, were tested for their ability to detect varying degrees of CAN, including any presence of CAN, early-stage CAN, and manifest CAN. The ROC are described in detail in **Table 3** and graphically in **Figure 2**. In general, the long-term mean difference performed poorly with all models, and early-stage CAN was difficult to detect with all CVT parameters. These models contain 0.5 in the confidence interval for the AUC, which means that random guessing cannot be ruled out. However, both short-term and long-term mean CVT performed better at detecting any degree of CAN and manifest CAN with AUCs in the range from 0.64-0.79, with a good balance between specificity and sensitivity, and high negative predictive values. These models suggest a cut-off of 2.7 LVS for short-term measured mean CVT and 5.0 LVS for long-term mean CVT. An alternative cut-off for long-term mean CVT is 7.5 LVS, but this results in a decrease in specificity.

**Table 3:** Receiver operating characteristics for cardiac vagal tone measures.

	CVT	AUC (95% CI)	FPR	TPR	Specificity (95% CI)	Sensitivity (95% CI)	PPV	NPV	Cut-off
CAN	Short-term mean	0.70 (0.54; 0.86)	0.28	0.68	0.71 (0.53; 0.85)	0.68 (0.52; 0.86)	0.65	0.75	2.7
	Long-term mean (1)	0.69 (0.54; 0.84)	0.25	0.56	0.75 (0.56; 0.87)	0.63 (0.38; 0.78)	0.63	0.72	5.0
	Long-term mean (2)	0.69 (0.54; 0.84)	0.50	0.85	0.50 (0.32; 0.67)	0.85 (0.63; 0.94)	0.54	0.82	7.5
	Long-term capacity	0.56 (0.39; 0.74)	0.29	0.55	0.71 (0.52; 0.86)	0.55 (0.26; 0.74)	0.57	0.68	11.7
Early CAN	Short-term mean	0.66 (0.48; 0.83)	0.64	1.00	0.36 (0.20; 0.55)	1.00 (0.77; 1.0)	0.45	1.00	4.2
	Long-term mean	0.64 (0.47; 0.82)	0.50	0.84	0.5 (0.32;0.67)	0.84 (0.58; 0.96)	0.44	0.88	7.5
	Long-term capacity	0.53 (0.32; 0.73)	0.28	0.46	0.71 (0.52; 0.84)	0.46 (0.23; 0.70)	0.42	0.74	11.7
Manifest CAN	Short-term mean	0.79 (0.59; 1.00)	0.28	0.83	0.72 (0.52; 0.86)	0.83 (0.43; 0.97)	0.42	0.95	2.7
	Long-term mean	0.78 (0.57; 0.99)	0.25	0.86	0.75 (0.57; 0.87)	0.86 (0.48; 0.97)	0.46	0.95	5.0
	Long-term capacity	0.64 (0.39; 0.90)	0.25	0.71	0.75 (0.56; 0.87)	0.71 (0.36; 0.91)	0.42	0.91	10.6

Area under the curve, sensitivity, and specificity are presented with 95% confidence intervals (CI). Long-term mean CVT yielded two long-models for CAN with the same Youden Index, marked (1) and (2). AUC: area under the curve, FPR: false positive rate, TPR: true positive rate, PPV: positive predictive value, NPV: negative predictive value. Number of participants: short-term *N* = 45; long-term *N* = 48.

## 4 Discussion

This feasibility study demonstrated the successful use of a novel non-contact capacitive ECG method, the *ProCVT* smart-sheet, for home-based recordings without contact surface electrodes. The sheet performed reasonably well with robust measures across multiple nights, and as such, the innovation meets technical requirements. The CVT level was lower in individuals with T2D, and associated with varying stages of CAN. Furthermore, CVT showed potential as a biomarker for CAN, effectively distinguishing individuals with any or manifest CAN from those without. However, long-term CVT recordings did not outperform short-term measures in predicting CAN stages.

Traditionally, long-term ECG recordings are performed using Holter monitors – a small device worn around the neck and connected to chest electrodes. However, wearing them for several days can be cumbersome, with tangled cables (“cable spaghetti”) prone to movement-induced artifacts. Single-channel ECG patches like the ePatch offer a cable-free alternative for up to five days of monitoring, but issues such as skin irritation and motion artifacts remain common. These limitations can reduce patient compliance with long-term monitoring. Many newer wireless systems are patches, handheld devices, or use Bluetooth in combination with traditional surface electrodes (24). In contrast, the *ProCVT* smart-sheet was developed to mimic a multiple-lead system with its six electrodes without the issue of the ‘cable spaghetti’ or skin irritation. However, as a wireless system without traditional skin electrodes, the device’s data quality required validation. We showed a signal quality of 13 or higher on average, constituting 93% of a recording, which is deemed very acceptable. While 21% of the data was missing before processing, this likely reflects individual sleep patterns, such as rolling off the sheet. Still, 5 hours of ECG data is generally sufficient to get an in-depth analysis of the autonomic tone. Data processing excluded 36 recordings due to a strict, conservative approach chosen to minimize artifacts common in ECG signals.

ECG is vital for assessing cardiac function and is increasingly available through consumer wearables, many of which track heart rate variability (HRV). HRV reflects the variation between successive R-R intervals, making its accuracy dependent on the precision of R-peak detection. Low resolution (8-10-bit) can mask low amplitude R-peaks or distort timing, whereas higher bit depth reduces rounding errors during analogue to digital conversion. While some studies suggest that a sampling rate of 25-250 Hz is sufficient for HRV analysis (25-28), an analytical model shows that at least 1000 Hz is needed for acceptable accuracy (29). This highlights the importance of high sampling rates and resolution for diagnostic purposes, and wearables cannot yet compete with medical-grade devices. The *ProCVT* smart-sheet combines a 1000 Hz sampling rate with 24-bit resolution, providing an interpolated precision of ~100-200 µs. This makes it well-suited for assessing CVT and HRV metrics such as the Root Mean Square of Successive Differences (RMSSD) and the high-frequency band i.e., RR-interval fluctuations in the 0.15-0.4 Hz range. These reflect parasympathetic activity, where it is necessary to detect changes of 50-20 ms in R-R intervals, and even a 5-ms error in R-peak timing can distort the values and possibly underestimate vagal activity. The combination of a home-monitoring system with high sampling rate, high resolution, multiple electrodes, and automatic upload makes the system well-suited for both clinical and research applications. Particularly the large size of the electrodes makes it very suitable for sleep measures, as it allows the patients to move freely while maintaining contact with the electrodes. This reduces the amount missing data and enhances patient compliance.

HRV includes non-linear, time-, and frequency-domain measures, amounting to around 25 parameters, which can complicate its clinical use. In contrast, CVT offers a simpler and more intuitive alternative. This study found a reduced CVT in T2D as well as in those with any degree of CAN and manifest CAN. These results are consistent with findings from Brock et al. (2017) and Wegeberg et al. (2020), conducted in individuals with type 1 diabetes (16, 17). However, this study found no significant difference in CVT between Early CAN and No CAN, limiting its usefulness for early detection. Both groups showed considerable variation in CVT, which may suggest less parasympathetic withdrawal than expected, potentially challenging current pathophysiological theories. However, it also shows that there is a considerable individual variation in CVT. When comparing CVT across multiple nights, 91% of the variance was attributed to differences between individuals, while only 9% was due to night-to-night variation. This suggests that tracking deviations from an individual’s baseline – rather than relying on a fixed cut-off – may be more informative, especially in relation to factors like stress, sleep quality, or neuropathy. The low night-to-night variation demonstrates that CVT is a reliable marker of parasympathetic tone, likely due to the recordings being taken at home during sleep, a setting shown to provide more robust measures of the heart’s adaptability (30). One advantage of CVT over HRV is its insensitivity to the use of beta blockers. Beta blockers reduce sympathetic activity and subsequently prolong the RR intervals, which can artificially inflate HRV measures (31). In contrast, CVT remains robust under these conditions, as it reflects dynamic vagal modulation, rather than absolute RR-interval duration. Since beta blockers tend to prolong RR intervals uniformly, they do not affect the variability captured by CVT.

Heart rate variability (HRV) is increasingly applied to classify sleep phases, as it is far more accessible than polysomnographic recordings. HRV is fairly effective in distinguishing rapid eye movement (REM) from non-REM sleep, since REM sleep is characterized by higher sympathetic tone (i.e., higher heart rate) and consequently lower parasympathetic tone (i.e., lower CVT) (32–34). This demonstrates the dynamic vagal modulation that occurs during sleep, which we aimed to capture with the concept of ‘CVT capacity’. During our data processing, some sleep segments with either high or low CVT may be excluded due to poor signal quality or artifacts, which carries the risk of under- or overestimating mean CVT. The conservative approach used in this study, requiring that at least 70% of the original data be preserved, with the majority of recordings exceeding 90%, minimizes this risk, while ensuring reliable representation of dynamic vagal modulation across sleep stages. CAN has previously been linked to impaired sleep outcomes (35, 36), probably due to disruption of the sleep architecture, with reduced non-REM sleep, due to parasympathetic withdrawal and sympathetic dominance. Parasympathetic withdrawal would be reflected as a lower mean CVT. However, polysomnographic confirmation of sleep phases is necessary to establish the relationship between CVT and REM/non-REM sleep. Nevertheless, imbalances in the distribution of non-REM and REM sleep are likely reflected in mean CVT, as has been consistently shown in HRV studies (32–34). In healthy individuals, the suppression of deep non-REM sleep reduces glucose tolerance (37). Thus, CAN may not only reduce sleep quality, but also exacerbate nocturnal hyperglycaemia, potentially aggravating pre-existing autonomic neuropathy.

This study investigated the ability of different CVT parameters to distinguish various degrees of CAN. Short-term and long-term mean CVT showed the strongest performance for detecting both the degree of CAN and manifest CAN, with AUCs ranging from 0.64-0.79, balanced sensitivity and specificity, and high negative predictive values – an important feature for screening. For manifest CAN, optimal cut-off points were 2.7 LVS (short-term) and 5.0 LVS (long-term), aligning with Wegeberg et al., 2020, who proposed a short-term threshold of 3.2 LVS in type 1 diabetes based on 5-minute recordings. For early CAN, this study found cut-offs of 4.2 (short-term) and 7.5 (long-term), close to Wegeberg et al.’s 5.2 (17). While we could not confirm CVT’s utility in detecting early CAN, the similar thresholds suggest comparable underlying pathophysiology in both cohorts. Furthermore, the higher long-term cut-point likely reflects increased parasympathetic activity during sleep in a home environment, consistent with known day-night differences in HRV (38). Moreover, a disadvantage of the 5-minute recording is that the CVT may be influenced by the surrounding area, and the patient may be less comfortable in clinical setting which could result in a lower CVT. Whereas the *ProCVT* smart-sheet can provide a more reliable estimate of the patient’s parasympathetic capacity due to the home element. Additionally, the technology used in the 5-minute recording does not offer real-time recording, requiring uploading and processing, and the processing takes the same amount of time whether the recording is five minutes or eight hours long. The *ProCVT* smart-sheet, however, automatically uploads data and has the potential for implementing automatic processing, reducing clinical workload and improving feasibility. The *ProCVT* smart-sheet also supports real-time recording, making it suitable not only for long-term monitoring but also for short-term recordings.

Furthermore, mean CVT outperformed CVT capacity and showed minimal variation across multiple nights, eliminating the need to select a single recording subjectively. This simplifies clinical use, requiring less specialized knowledge. Surprisingly, short- and long-term mean CVT demonstrated similar performance despite being recorded on different devices, suggesting the robustness of the underlying algorithm. Both approaches may be suitable for evaluating CAN in individuals with diabetes, though further studies are needed to explore their ability to detect early-stage CAN, where intervention is still possible. When considering clinical implementation, health economic factors are important. Both methods require high-resolution ECG devices, which are costlier than standard equipment but differ in operational demands. The short-term method involves healthcare staff and yields immediate results, while the smart-sheet allows for patient-led long-term recordings at home, though data must be processed afterward. Ultimately, the choice of method should align with clinical workflows, staff capacity, and patient preferences.

While cross-sectional studies offer valuable insights, they also have inherent limitations. Although this study shows that CVT remains stable over multiple nights, a longitudinal design would better assess its ability to track CAN progression through declining CVT. We excluded recordings from the *ProCVT* smart-sheet if less than 70% of the original data remained. While this may seem stringent, we opted for a conservative approach to minimize entire sleep phases being removed and thus ensure reliable cut-off point determination We acknowledge that the selection of the ‘best’ recording introduces bias; however, further analysis showed limited night-to-night variation in mean CVT suggesting that this only had a small influence on the cut-off values. Additionally, the study included 40 participants with T2D, most without signs of CAN. Future research should involve a larger, more balanced cohort across CAN stages, particularly early and manifest CAN, to improve understanding of CVT dynamics. Given the predominance of the No CAN group, a 2:1 control-to-case ratio is recommended. Based on a target AUC of 0.75, 80% power, and a 5% significance level, at least 19 early CAN cases and 38 controls are needed in future studies to ensure adequate statistical power. Moreover, it is a limitation that, without polysomnographic confirmation of sleep phases, phase-specific central–autonomic nervous system interactions could not be explored. Unfortunately, polysomnography was not feasible in the home setting of the present study.

## 5 Conclusion

This preliminary study demonstrated that the novel *ProCVT* smart-sheet is a feasible non-invasive method for obtaining ECG recordings effortlessly without surface electrodes, easing the integration of digital medical devices in T2D care. CVT emerged as a reliable biomarker for CAN, effectively distinguishing individuals with any degree or manifest CAN from those without. Long-term recordings were not superior to short-term recordings, suggesting both are viable options. With further development, especially in early-stage CAN detection, the *ProCVT* smart-sheet could become a valuable clinical tool. Its robust technology may also hold potential for broader use in other neurodegenerative conditions involving autonomic dysfunction, pending further validation.

## Data Availability

The data supporting the conclusions of this article will be made available on reasonable request.
